# Veterinary Association of the District of Columbia

**Published:** 1897-09

**Authors:** J. P. Turner

**Affiliations:** Secretary


### VETERINARY ASSOCIATION OF THE DISTRICT OF COLUMBIA.

THE fourteenth meeting of the Association was held at Elks' Hall, No. 1006 E Street, N. W., July 31st, at 8 P.M. In the absence of the President, the Vice-President, Dr. Buckingham, called the meeting to order. The following members answered the roll-call: Drs. Barton, Buckingham, Turner, Walmer, and Robinson, C. B. Dr. Yetton, an applicant for membership, was also present. A letter was received from Dr. C. F. Hadfield expressing regrets that he would be unable to attend the meeting.

The Committee on Credentials made a favorable report on the applications of Drs. Salmon and Yetton for membership, and on motion of Dr. Robinson they were elected members of this Association.

The Legislative Committee reported that no active measures had been taken regarding the bill to regulate the practice of veterinary medicine in the District of Columbia at the extra session of the Fifty-fifth Congress, for obvious reasons, but that a vigorous effort would be made at the coming session in December.

The subject of rabies and its existence in the District was discussed in its many phases, and the Association resolved itself into a committee of the whole, and drafted the following resolutions, which were ordered to be forwarded to the Commissioners of the District of Columbia:

WHEREAS, This Association recognizes rabies as a disease, and that it exists at present in the District of Columbia among the lower animals and man, and that we as a body have recognized this enzoötic disease in a large number of animals, particularly dogs;

WHEREAS, We believe this disease is increasing in this District, and while we recognize that there is no cure for it after the disease is developed, we firmly believe that the disease can be prevented by proper sanitary measures; therefore, be it

*Resolved*, That this Association recommends to the Honorable Commissioners of the District of Columbia that all dogs running at large during the months of June, July, and August, be required to wear a proper muzzle, and that all unmuzzled dogs be considered dangerous to the health and welfare of the public, and that they be treated as unlicensed dogs and impounded, and that a copy of these resolutions be forwarded to the Honorable Commissioners of the District of Columbia.

Dr. Robinson reported a very interesting case of rabies affecting a mule. The points of interest in the case were the vicious attacks by the teeth made on anything which it encountered, and the fact that the animal moved in a straight line from one side of the corral to the other.

Dr. Buckingham cited several cases of rabies which had existed among the wolves at the Zoological Garden.

Dr. Barton reported several cases of dumb rabies.

For the next meeting (September 25th) it was agreed that the subject of tuberculosis should be discussed in its various forms, each member discussing it in one of its peculiar relations to the public health.

The meeting was then adjourned.

J. P. TURNER,
Secretary.

